# Congenital Bladder Diverticulum Presenting as Urinary Retention in a Neonate: A Case Report

**DOI:** 10.7759/cureus.82862

**Published:** 2025-04-23

**Authors:** Claudia Berrondo

**Affiliations:** 1 Surgery/Pediatric Urology, University of Nebraska Medical Center, Omaha, USA; 2 Pediatric Urology, Children’s Nebraska, Omaha, USA

**Keywords:** clean intermittent catheterization, congenital bladder diverticulum, diverticulectomy, neonate, urinary retention

## Abstract

Congenital bladder diverticulum is a rare cause of acute urinary retention in infants and children, often requiring surgical repair. Given technical complexities and anesthetic risks associated with neonatal surgery, neonates presenting with urinary retention are typically managed using a staged approach involving an immediate vesicostomy followed by delayed surgical repair. We present the case of a two-week-old male neonate with acute urinary retention due to a large congenital bladder diverticulum. His urinary retention was initially managed with clean intermittent catheterization (CIC). He ultimately underwent successful surgical excision of the diverticulum at seven months of age. Initial management with CIC may obviate the need for immediate surgery in neonates and young infants with congenital bladder diverticula presenting with acute urinary retention.

## Introduction

Bladder diverticulum, defined as a hernia of bladder urothelium through the muscle wall, is a rare condition. Bladder diverticula can be categorized as primary (congenital) or secondary, with most being secondary in origin. In infants and children, most secondary bladder diverticula are due to urethral obstruction (such as posterior urethral valves (PUV)), neurogenic bladder, or connective tissue disorders. Congenital bladder diverticula are rare, with an estimated incidence of 1.7%, and are more commonly diagnosed in males [1,2]. They are thought to develop due to weakness in the detrusor muscle, usually at or near the ureterovesical junction [1]. 

Vesicoureteral reflux (VUR) may occur when the ureterovesical junction becomes incorporated into the bladder diverticulum as it enlarges [2,3]. Most small diverticula are asymptomatic and are identified incidentally. These small, clinically insignificant diverticula do not require monitoring or treatment. As diverticula enlarge, they can lead to a variety of clinical presentations and ultimately require surgical treatment. The most common presenting symptoms include hematuria, VUR, or sequelae of urinary stasis such as urinary tract infections due to bacterial overgrowth in the stagnant urine, and bladder stones as minerals in the urine precipitate and accumulate within the diverticulum [2-4].

Obstructive urinary symptoms and urinary retention are rarely reported symptoms in pediatric patients with congenital bladder diverticula. These symptoms are even rarer in neonates, with limited cases reported in the literature [3-9]. While the mechanism remains poorly understood, bladder diverticula are believed to cause bladder outlet obstruction through mechanical means, either compression and/or kinking of the bladder outlet or urethra [5,8]. Recognizing the inherent technical challenges associated with performing surgical excision in neonates, the current medical consensus, as reflected in the literature, favors a staged management approach. This strategy involves initial urinary diversion with a vesicostomy for immediate treatment of the urinary retention, followed by delayed definitive repair with excision of the diverticulum [2,3,5].

We report a case of a two-week-old male neonate with urinary retention secondary to a large congenital bladder diverticulum, initially managed successfully with clean intermittent catheterization (CIC), with delayed definitive surgical treatment at seven months of age.

## Case presentation

**Figure 1:**
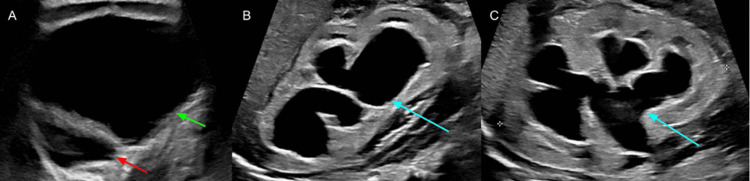
Renal and bladder ultrasound at presentation (A) Transverse view demonstrating a distended bladder (green arrow) and bladder diverticulum (red arrow). (B) Sagittal view of the right kidney demonstrating hydronephrosis (blue arrow). (C) Sagittal view of the left kidney demonstrating hydronephrosis (blue arrow).

A two-week-old male neonate presented to the emergency room with a distended abdomen. Abdominal ultrasound (US) demonstrated a 10.5 cm cystic abdominal mass with bilateral hydroureteronephrosis (Figure 1).

**Figure 2:**
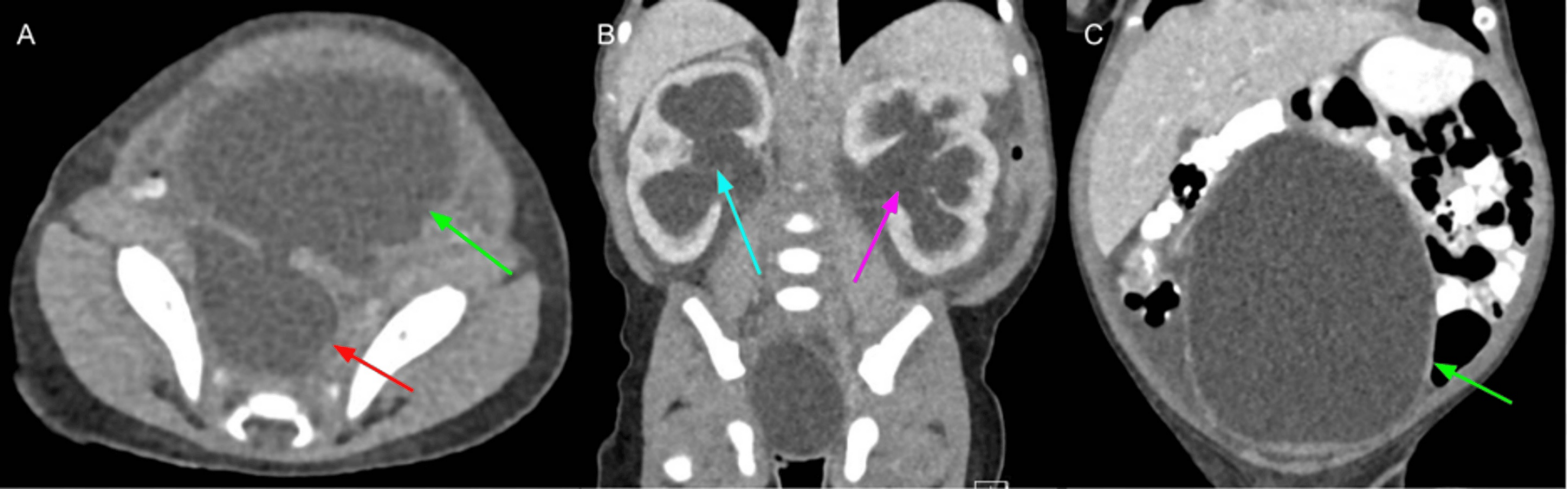
Computed tomography of the abdomen and pelvis at presentation (A) Transverse view demonstrating a distended bladder (green arrow) and bladder diverticulum (red arrow). (B) Coronal view demonstrating hydronephrosis of the right kidney (blue arrow) and hydronephrosis of the left kidney (purple arrow). (C) Coronal view demonstrating a massively distended bladder (green arrow).

Initial creatinine was elevated at 1.96 mg/dL (reference range 0.25-0.65 mg/dL), and he was hyponatremic with a serum sodium of 127 mmol/L (reference range 135-145 mmol/L). Due to the concern for a possible enteric duplication, sacrococcygeal teratoma, anterior myelomeningocele, or neuroblastoma, he underwent a computed tomography (CT) scan of the abdomen and pelvis, which revealed a severely distended bladder and bilateral hydroureteronephrosis with renal parenchymal thinning and a large right periureteric diverticulum (Figure 2).

A urethral catheter was placed, and a follow-up renal and bladder US demonstrated improved hydronephrosis and a decrease in the size of the diverticulum. He developed post-obstructive diuresis, which was treated with intravenous fluid replacement. His creatinine improved to 0.23 mg/dL, and his hyponatremia resolved (Table 1).

**Table 1:** Serum creatinine and sodium levels

	Day 1	Day 1	Day 2	Day 2	Day 2	Day 2	Day 2
Creatinine (reference range 0.25-0.65 mg/dL)	1.96 mg/dL	1.55 mg/dL	0.37 mg/dL	0.27 mg/dL	0.22 mg/dL	0.24 mg/dL	0.23 mg/dL
Sodium (reference range 135-145 mmol/L mmol/L)	127 mmol/L	134 mmol/L	146 mmol/L	144 mmol/L	139 mmol/L	136 mmol/L	135 mmol/L

**Figure 3:**
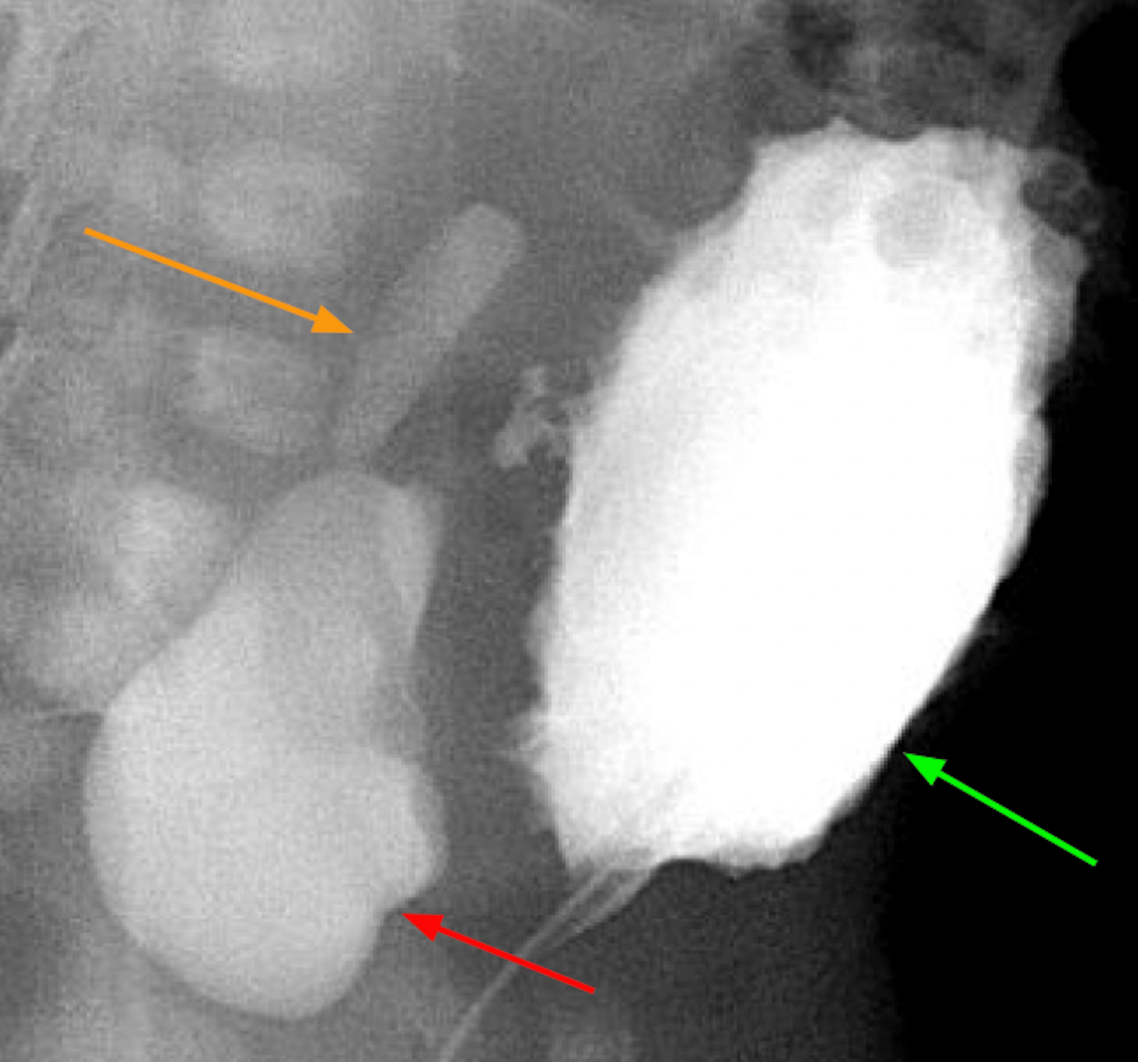
Voiding cystoureterogram demonstrating a trabeculated bladder (green arrow), large bladder diverticulum (red arrow) with reflux into the right distal ureter (orange arrow)

A voiding cystourethrogram (VCUG) demonstrated a trabeculated bladder and a large right periureteric diverticulum with grade 1 VUR into the distal right ureter (Figure 3).

A discussion was held with the parents regarding the management of urinary retention. Ultimately, the family chose CIC over vesicostomy to avoid general anesthesia in the neonatal period. He was transitioned to CIC and discharged home. A follow-up renal and bladder US four weeks later demonstrated improved hydroureteronephrosis and decreased dilation of the diverticulum. His parents performed CIC four to five times per day, which he tolerated well. The patient remained free of urinary tract infections and was not on antibiotic prophylaxis. Periodic monitoring showed stable, normal creatinine. 

**Figure 4:**
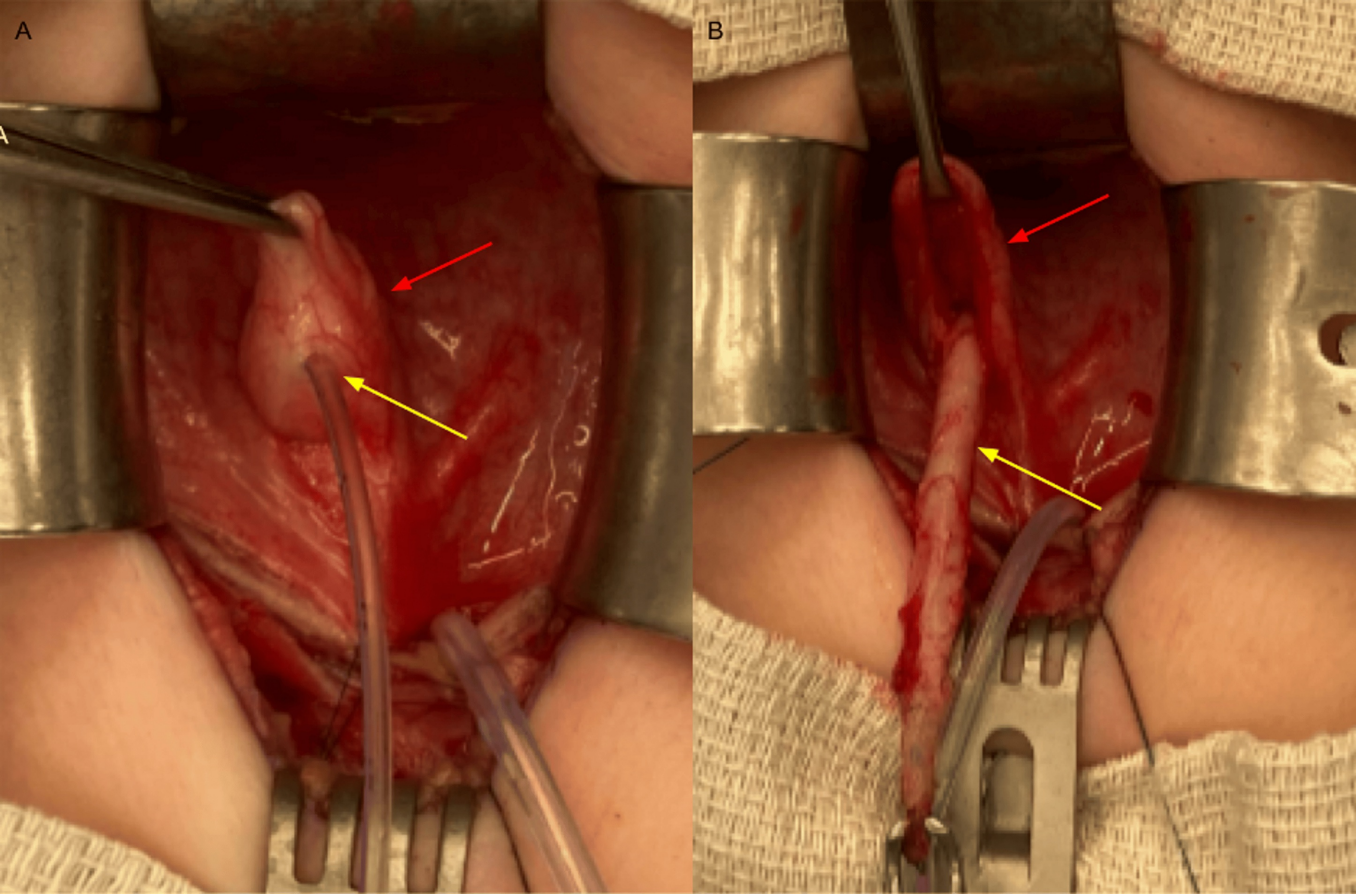
Intraoperative photos (A) Right ureteral orifice (yellow arrow) within the everted diverticulum (red arrow). (B) Right ureter (yellow arrow) dissected from within the diverticulum (red arrow).

At seven months of age, he underwent surgical repair with an open diverticulectomy and right ureteral reimplantation. Cystourethroscopy revealed a normal urethra and a trabeculated bladder. Inspection of the trigone revealed a normal and orthotopic left ureteral orifice and a large diverticulum in the expected location of the right ureteral orifice. The right ureteral orifice was not visualized. The bladder was approached through a Pfannenstiel incision. An anterior cystotomy was performed, and the diverticulum was identified on the right side of the trigone. The diverticulum was everted, and the right ureteral orifice was identified within the diverticulum (Figure 4).

The diverticulum was fully excised via an intravesical approach. The right vas deferens was extremely adherent to the diverticulum and could not be separated. A small section of the vas deferens was excised along with the diverticulum. A vasovasostomy was then performed using 10-0 nylon sutures. The muscular defect in the bladder was closed, and then the right ureter was reimplanted using a modified Leadbetter-Politano technique. His postoperative course was uneventful. His urethral catheter was removed on postoperative day one; he was able to void spontaneously, and he was discharged home.

**Figure 5:**
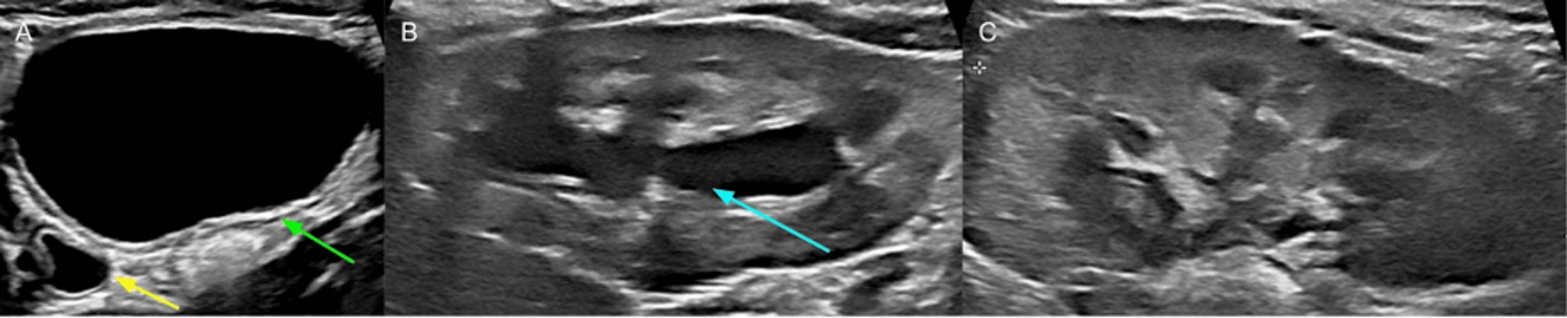
Renal and bladder ultrasound one month postoperatively (A) Transverse view demonstrating a full bladder (green arrow) and distal right ureteral dilation (yellow arrow). (B) Sagittal view of the right kidney demonstrating improved hydronephrosis (blue arrow). (C) Sagittal view of the left kidney demonstrating resolution of the hydronephrosis.

At one month postoperatively, he continued to void spontaneously. A renal and bladder US demonstrated a normal left kidney and significantly improved right hydronephrosis with right distal ureteral dilation (Figure 5).

**Figure 6:**
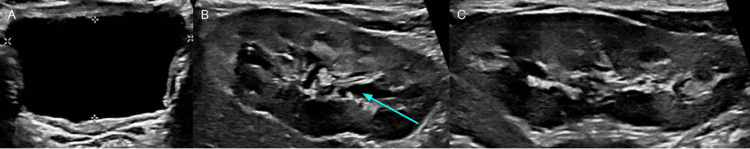
Renal and bladder ultrasound eight months postoperatively (A) Transverse view demonstrating a full bladder. (B) Sagittal view of the right kidney demonstrating minimal hydronephrosis (blue arrow). (C) Sagittal view of the left kidney demonstrating complete resolution of the hydronephrosis.

At eight months postoperatively, he continued to void spontaneously and was doing well clinically without urinary tract infections or other urinary symptoms. His renal and bladder US demonstrated further improvement of right hydronephrosis and resolution of distal ureteral dilation (Figure 6). 

## Discussion

Bladder diverticula in children can be categorized as congenital or acquired. Acquired diverticula develop secondary to obstructive diseases such as PUV, connective tissue disorders, or neurogenic bladder [2,7,8]. Congenital bladder diverticulum is a rare congenital anomaly with an estimated incidence of 1.7% and primarily affects males [1,10]. The etiology is due to weakness in the detrusor muscle in the trigone near the ureteral orifice, most commonly lateral and superior to the ureteral orifice. As the diverticulum enlarges, the ureter may be incorporated into it [4,8]. They are mostly asymptomatic, although patients with large diverticula may present with hematuria or urinary tract infections due to urinary stasis in the diverticulum [1,6]. Small asymptomatic diverticula do not require treatment; however, symptomatic diverticula require surgical repair. Surgical indications include persistent or recurrent UTI, bladder stones, obstructive symptoms (including urinary retention), and/or VUR [2,8,10].

Urinary retention is a rare presenting symptom of congenital bladder diverticula, especially in neonates [3-7]. Urinary retention is thought to be the result of mechanical compression and/or angulation of the bladder neck and/or urethra as the diverticulum enlarges [5,8]. There are limited reports of pediatric patients with obstructive urinary symptoms or acute urinary retention due to congenital bladder diverticula. Aslam et al. reported an 11-month-old male who initially presented with an abdominal mass and obstructive voiding symptoms due to a large diverticulum [4]. At that time, the family chose not to pursue surgical intervention. Three months later, the patient developed sepsis and acute urinary retention and was successfully treated with diverticulectomy and ureteral reimplantation. Oge et al. report a case of a 2.5-year-old male presenting with urinary retention and a palpable abdominal mass secondary to a large bladder diverticulum [6]. This patient was successfully treated with diverticulectomy and ureteral reimplantation. Shukla et al. reported a case series of four patients (three male and one female) with bladder outlet obstruction secondary to large bladder diverticula presenting at four months to 11 years of age [7]. All patients presented with obstructive symptoms (slow urine stream, incomplete emptying), but none developed urinary retention. All were treated with diverticulectomy, with three of the four requiring ureteral reimplantation. Bhat et al. reported a series of 12 pediatric patients presenting with urinary retention secondary to primary bladder diverticula who underwent surgical repair [2]. Six of the 12 patients in their cohort were under one year of age, with the youngest patient being one month old.

Surgical excision is technically challenging in neonates, and the current recommendation in the literature is to utilize a staged approach with urgent vesicostomy followed by delayed surgical repair, typically after one year of age [3,5]. Bogdanos et al. reported a series of 22 children with large diverticula requiring surgical intervention [3]. Two of the 22 patients were neonates presenting with urinary retention managed initially with vesicostomy, followed by excision after one year of age. Singal et al. reported a series of infants presenting with poor urinary stream or urinary retention secondary to a congenital bladder diverticulum [5]. Two of the seven patients were neonates managed with vesicostomy with a plan for definitive surgery at 1 year of age. 

Although there are reports of young infants with urinary retention due to congenital bladder diverticula managed with immediate surgical repair, current recommendations in the literature are to manage urinary retention in neonates using urinary diversion with vesicostomy, followed by delayed surgical repair after one year of age. To our knowledge, this is the first reported case of a neonate presenting with urinary retention secondary to a congenital bladder diverticulum successfully managed initially with CIC followed by definitive surgical repair. The use of CIC allowed for successful non-operative management in the neonatal period.

## Conclusions

A congenital bladder diverticulum, defined as a primary outpouching of the bladder mucosa through the bladder muscle, is a very rare cause of urinary retention in pediatric patients. The mechanism by which a congenital bladder diverticulum leads to urinary retention is believed to involve mechanical compression and/or angulation as the diverticulum progressively enlarges. CIC offers a safe and viable alternative to vesicostomy for the management of urinary retention in neonates with congenital bladder diverticula until definitive surgical repair can be safely undertaken.
